# P-1204. Hospitalizations Associated with Respiratory Syncytial Virus (RSV) Illness Among Children and Adolescents in Ontario, Canada

**DOI:** 10.1093/ofid/ofae631.1386

**Published:** 2025-01-29

**Authors:** Sazini Nzula, Alexandra Goyette, Deshayne B Fell, Ceryl Tan, Natalie Nightingale, Maria Esther Perez Trejo, Calum S Neish, Ana Gabriela Grajales

**Affiliations:** Pfizer Canada, Kirkland, Quebec, Canada; Pfizer Canada, Kirkland, Quebec, Canada; Pfizer Inc., New York City, New York; IQVIA Solutions Canada Inc, Mississauga, Ontario, Canada; IQVIA Solutions Canada Inc, Mississauga, Ontario, Canada; IQVIA Solutions Canada Inc, Mississauga, Ontario, Canada; IQVIA Solutions Canada Inc, Mississauga, Ontario, Canada; Pfizer Canada ULC, Kirkland, Quebec, Canada

## Abstract

**Background:**

Respiratory syncytial virus (RSV) is a leading cause of lower respiratory tract illness (LRTI) among children, particularly in young infants. Although the burden of illness is higher at a younger age, it has been less documented in children 5-17 years old.

**Figure:**
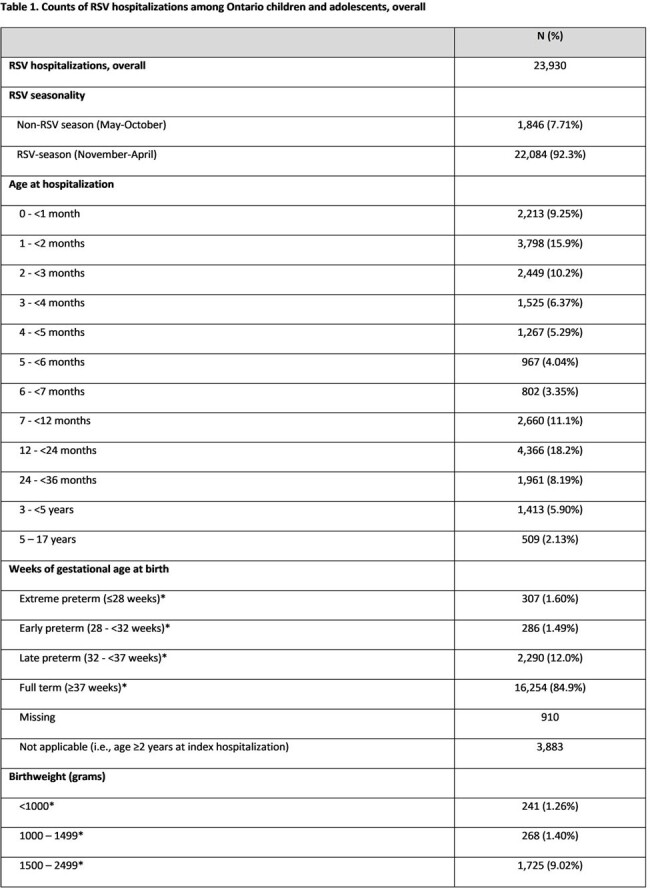

*Missing and Not Applicable categories not considered while calculating %

#Risk status includes conditions such as congenital malformations, cystic fibrosis, chronic respiratory disease, trisomy 21, cardiovascular disorders, congenital kidney disease (in < 2 years only), congenital neurological disorders (in < 2 years only), cancer (in 2-17 years only), immunodeficiencies (in 2-17 years only), and neuromuscular diseases (in 2-17 years only)

RSV: respiratory syncytial virus; wGA: weeks of gestational age

**Methods:**

Patients aged ≤ 17 years hospitalized with RSV between July 1^st^, 2010 and March 31^st^, 2023 were identified from provincial administrative data at ICES, which captures all healthcare encounters within Ontario’s publicly funded healthcare system. Annual outcomes were reported from July 1^st^ to June 30^th^ of the next year.

**Figure 1.**
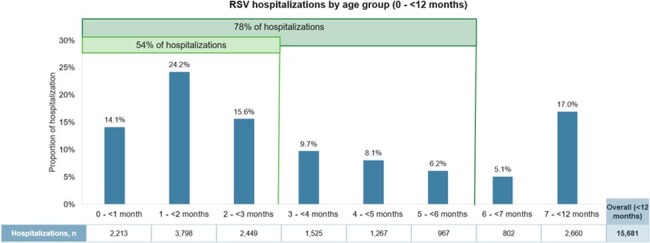
RSV hospitalizations among Ontario children < 12 months old (2010 – 2023). Note that the age groups are not consistent in size (i.e., 1-month groups for those < 7 months while 7 - < 12 months were all grouped together).

**Results:**

Overall, 23,930 RSV hospitalizations were reported; annual counts ranged between 1,356 (2010-11) and 4,298 (2022-23) (**Table 1**). Children < 12 months accounted for 66% of all hospitalizations, of which > 75% occurred in those < 6 months (**Figure 1**), and over 90% of hospitalizations occurred during the RSV-season (November-April). Although children < 2 years made up a greater proportion of hospitalizations compared to those 2-17 years (84% vs. 16%), risk conditions were more common in those 2-17 years compared to < 2 years of age (35% vs. 7%). The median length of stay (LOS) per RSV hospitalization was 69 hours (h). Longer LOS was observed for those hospitalized at age < 1 month (108 h), 5-17 years (92 h), born preterm (84-115 h), with a low birthweight (87-119 h), or with a risk condition (92-97 h). About 12% of the cohort had an intensive care unit (ICU) stay (median LOS: 87 h), which made up 55% of their LOS in the hospital. A greater proportion of those hospitalized at < 4 months old (16%) or 5-17 years old (22%) had an ICU stay. While the proportion of patients with an ICU stay increased from 8% in 2010-11 to 17% in 2022-23, the median LOS in ICU decreased from 101 h to 78 h over the same period. Overall, in-hospital, all-cause mortality was 0.12%, but higher for those with risk conditions (< 2 years: 0.61%; 2-17 years: 1.02%).

**Conclusion:**

This study highlighted those who may be more impacted by RSV illness: younger infants, those born prematurely or with a low birthweight, and with underlying risk conditions. While most hospitalizations were observed in those < 12 months, RSV remains a noteworthy cause of hospitalization in older children, especially those with risk conditions.

**Disclosures:**

**Sazini Nzula, PhD**, Pfizer Canada: Employee **Alexandra Goyette, MSc**, Pfizer: Employee|Pfizer: Stocks/Bonds (Private Company) **Deshayne B. Fell, PhD**, Pfizer Inc.: Employment|Pfizer Inc.: Stocks/Bonds (Private Company) **Ana Gabriela Grajales, MD**, Pfizer Canada ULC: I am currently an employee in Medical Affairs|Pfizer Canada ULC: Stocks/Bonds (Public Company)

